# Quantitative volumetric computed tomography embolic analysis, the Qanadli score, biomarkers, and clinical prognosis in patients with acute pulmonary embolism

**DOI:** 10.1038/s41598-022-11812-6

**Published:** 2022-05-10

**Authors:** Wei-Ming Huang, Wen-Jui Wu, Sheng-Hsiung Yang, Kuo-Tzu Sung, Ta-Chuan Hung, Chung-Lieh Hung, Chun-Ho Yun

**Affiliations:** 1 Department of Radiology, Mackay Memorial Hospital, Taipei City, 10449 Taiwan; 2 Department of Medicine, Mackay Medical College, New Taipei City, 25245 Taiwan; 3 Mackay Junior College of Medicine, Nursing, and Management, New Taipei City, 25245 Taiwan; 4 Division of Pulmonary and Critical Care Medicine, Mackay Memorial Hospital, Taipei City, 10449 Taiwan; 5 Cardiovascular Division, Department of Internal Medicine, Mackay Memorial Hospital, Taipei City, 10449 Taiwan; 6 Graduate Institute of Health Care Organization Administration, College of Public Health National Taiwan University, Taipei City, 100 Taiwan

**Keywords:** Cardiovascular diseases, Cardiovascular biology

## Abstract

Detailed descriptions of acute pulmonary emboli (PE) morphology, total embolic volume (TEV), and their effects upon patients’ clinical presentation and prognosis remain largely unexplored. We studied 201 subjects with acute PE to the emergency department of a single medical center from April 2009 to December 2014. Patient hemodynamics, Troponin I and D-dimer levels, echocardiography, and the 30-day, 90-day and long-term mortality were obtained. Contrast-enhanced computed tomography (CT) of pulmonary structures and 3-dimensional measures of embolic burden were performed. The results showed a linear association between the greater TEV and each of the following 4 variables (increasing incidence of right ventricular (RV) dysfunction, higher systolic pulmonary artery pressure (sPAP), greater RV diameter, and RV/left ventricular (LV) ratio (all *p* < 0.001)). Among the measures of CT and echocardiography, TEV and RV/LV ratio were significantly associated with impending shock. In backward stepwise logistic regression, TEV, age and respiratory rate remained independent associated with impending shock (OR: 1.58, 1.03, 1.18, respectively and all *p* < 0.005).Total embolic burden assessed by CT-based quantification serves as a useful index for stressed cardiopulmonary circulation condition and can provide insights into RV dysfunction and the prediction of impending shock.

## Introduction

Acute pulmonary embolism (PE) is a major public health concern and a potentially fatal disease. In the United States, the average incidence is about 0.1% per year, and more than 100,000 people die from acute PE annually^1^. Once a diagnosis of acute PE has been successfully established, patient risk stratification and prognostication are essential for guiding subsequent clinical management. The recent clinical guidelines published by the American Heart Association (AHA) and the European Society of Cardiology (ESC)^2,3^ stratify patients based on massive, submassive, and low-risk PE categories or the Pulmonary Embolism Severity Index (PESI)^4^, respectively. Both incorporate key prognostic indicators such as the level of hemodynamic stability, myocardial injury, and right ventricular dysfunction.

Rapid and accurate diagnosis is critical for managing patients presenting with acute PE, and over the last twenty years, multidetector computed tomography (MDCT) with high spatial resolution (1–2 mm) has become highly capable of detecting emboli within the main branches of the pulmonary arteries^5^. This has revolutionized the diagnostic approach and serves as the first-line imaging diagnostic tool for acute PE clinically^6^.

Interestingly, as the most commonly used diagnostic test for acute PE, MDCT can provide detailed information on embolic location and burden, neither of which is considered in the above guidelines to be a prognostic marker. Tuzovic et al. found that central and multilobar clots were correlated with right ventricle (RV) dysfunction^7^, but studies have not shown any significant correlation between clot burden, as measured using current two-dimensional (2D) methods, and adverse clinical outcomes^8,9^. In contrast, some studies have shown a correlation between a patient’s clot location and burden, as measured using either 2D methods or the Mastora or Qanadli scores, and their clinical condition^10,11^. Praveen et al. also found that a higher clot burden was associated with right heart strain and adverse clinical events^12^. Previously, measuring clot burden using three-dimensional (3D) methods was impractical because it requires a large amount of manpower during daily clinical practice. It is also debated that the role of clot burden as an indicator of short-term prognosis^13^. Recently, however, a 3D-based computed tomography (3D CT) method was developed, and it has been widely applied to oncology patient images for precise tumor measurement^14,15^. As a result of the advancement of computer science and software, this 3D CT method has become more accurate and less demanding. Therefore, we aimed to assess the association between clot volume burden estimated using this 3D CT method and the Qanadli score, biomarkers, and clinical outcomes, including impending shock (normotension at arrival and subsequent development of hypotension requiring vasopressor) and short-term mortality.

## Results

### Patient characteristics

The mean age of the patient population was 66.8 ± 17 years, and it included 122 females (60.7%) and 79 males (39.3%). Their characteristics are shown in Table 1. A minority of the patients had either a history of cancer or chronic heart/lung disease (23.9% and 45.1%, respectively), and the majority (79.3%) had a high-risk sPESI score. Echocardiographic RV dysfunction was present in 30.2% of the patients. The arrival vital signs were generally stable, with saturation > 90% (93.9 ± 5.6%), systolic blood pressure > 100 mmHg (128.6 ± 25.9 mmHg), and respiratory rate < 30 per min (21.9 ± 5.24 per min). The lab data shows that most of the patients had BNP ≤ 400 (72.1%) and troponin I ≤ 0.04 (55.2%) at arrival, with an average D-dimer of 10,363 ng/mL.

**Table 1:** Characteristics of the study population.

Age (years)	66.8 (± 17.0)
**Gender**	
Male	79 (39.3%)
Female	122 (60.7%)
**History of cancer**	
Yes	48 (23.9%)
No	153 (76.1%)
**History of chronic heart lung disease**	
Yes	88 (45.1%)
No	107 (54.9%)
**Arrival vital signs**	
SpO_2_ (%)	93.9 (± 5.6)
SBP	128.6 (± 25.9)
DBP	73.4 (± 16.4)
RR	21.9 (± 5.24)
**sPESI score**	
High risk (≥ 1)	142 (79.3%)
Low risk (< 1)	37 (20.7%)
**Lab data**	
BNP	
BNP > 400	39 (27.9%)
BNP ≤ 400	101 (72.1%)
**Troponin I**	
Troponin I > 0.04	81 (44.8%)
Troponin I ≤ 0.04	100 (55.2%)
D-dimer	10,363 (± 74,701)
**Echocardiogram data**	
sPAP	42.3 (± 16.8)
**RV dysfunction**	
Yes	42 (30.2%)
No	97 (69.8%)
**Mortality**	
30 days Mortality	17 (8.99%)
90 days Mortality	28 (14.8%)

*SBP* systolic blood pressure, *RR* respiratory rate, *sPAP* systolic pulmonary arterial pressure, *RV* right ventricle, *LV* left ventricle, *RV/LV* ratio of diameter of RV and diameter of LV.

### CT findings

The TEV was 8.5 ± 9.2 cm^3^, and the embolic volume in the right and left pulmonary arterial tree was 5.26 ± 5.96 cm^3^ and 3.17 ± 4.33 cm^3^ (Table 2). The mean time requirement of 3D segmentation using workstation for each case was 36.0 ± 10.0 min, including emboli detection and volume measurement. The overall Qanadli score was 6.8 ± 4.0. The average diameter of the RV was 4.50 ± 0.85 cm and the RV/LV ratio was 1.39 ± 0.55. The average diameter of main pulmonary artery (MPA) was 3.01 ± 0.49 cm.

**Table 2:** CT assessments of pulmonary emboli.

CT data	
Total emboli volume	8.5 (± 9.2) (cm^3^)
Qanadli score	6.8 (± 4.0)
Left side emboli volume	3.17 (± 4.33) (cm^3^)
Right side emboli volume	5.26 (± 5.96) (cm^3^)
Diameter of RV	4.50 (± 0.85) (cm)
RV/LV	1.39 (± 0.55)
MPA	3.01 (± 0.49) (cm)

*RV* right ventricle, *LV* left ventricle, *MPA* diameter of main pulmonary artery.

With increasing TEV quartile (Table 3), the patients exhibited increased RV dysfunction, higher sPAP, larger RV diameter, increased RV/LV ratio, larger MPA, and greater incidence of abnormal troponin I levels. Age, gender, and BNP were not related to TEV in this population. In addition, SpO_2_ decreased significantly with increasing TEV.

**Table 3:** Total emboli volume (TEV) quartiles.

	Q1 (n = 50)	Q2 (n = 50)	Q3 (n = 50)	Q4 (n = 51)	*p*-value
TEV (cm^3^)	0.44	2.49	8.07	21.78	
Age	68.06	68.74	64.40	66.06	0.337
Gender (male)	40%	34%	40%	43.1%	0.820
RV dysfunction	27.8%	6.06%	34.3%	51.4%	0.0007*
sPAP	38.39	39.08	45.51	46.44	0.0144*
Diameter of RV	4.19	4.20	4.54	5.05	< 0.001*
RV/LV	1.10	1.18	1.40	1.87	< 0.001*
MPA	2.9**3**	2.97	2.96	3.16	0.0239*
SpO_2_	96.44	93.03	93.81	92.30	0.0138*
BNP > 400	25.7%	17.6%	24.2%	42.1%	0.117
Troponin I > 0.04	29.8%	39.5%	41.9%	66.7%	0.0026*

*TEV* total embolic volume, *RV* right ventricle, *LV* left ventricle, *sPAP* systolic pulmonary artery pressure, *BNP* B-type natriuretic peptide, *MPA* diameter of main pulmonary artery. * The threshold for statistical significance was *p* < 0.05.

We used multivariate logistic regression analysis to evaluate the relationship between RV dysfunction and several clinical and radiologic factors (age, gender, systolic blood pressure [SBP], respiratory rate, history of cancer, history of chronic heart/lung disease, BNP, troponin I, and TEV). The TEV was expressed as unit of 10cm^3^. The backward stepwise selection method was used for model selection. The final model included TEV and troponin I, which provided a reasonable model fit (Table 4). TEV was significantly related to the incidence of RV dysfunction (OR = 1.93, *p* = 0.0039).

**Table 4:** Backward stepwise logistic regression for RV dysfunction.

	OR	95% CI	*p*-value
Total emboli volume (unit of 10 cm^3^)	1.93	1.25–3.09	0.0039*
Troponin I > 0.04	1.61	0.72–3.63	0.25

The Full model includes total emboli volume, chronic heart lung disease, age, gender, history of cancer, BNP, Troponin I, systolic blood pressure and respiratory rate as the predicting factors. After backward stepwise selection, the remaining factors were total emboli volume and tropoin I.

*BNP* B-type natriuretic peptide, *MPA* diameter of main pulmonary artery. * The threshold for statistical significance was* p* < 0.05.

**Figure 1:**
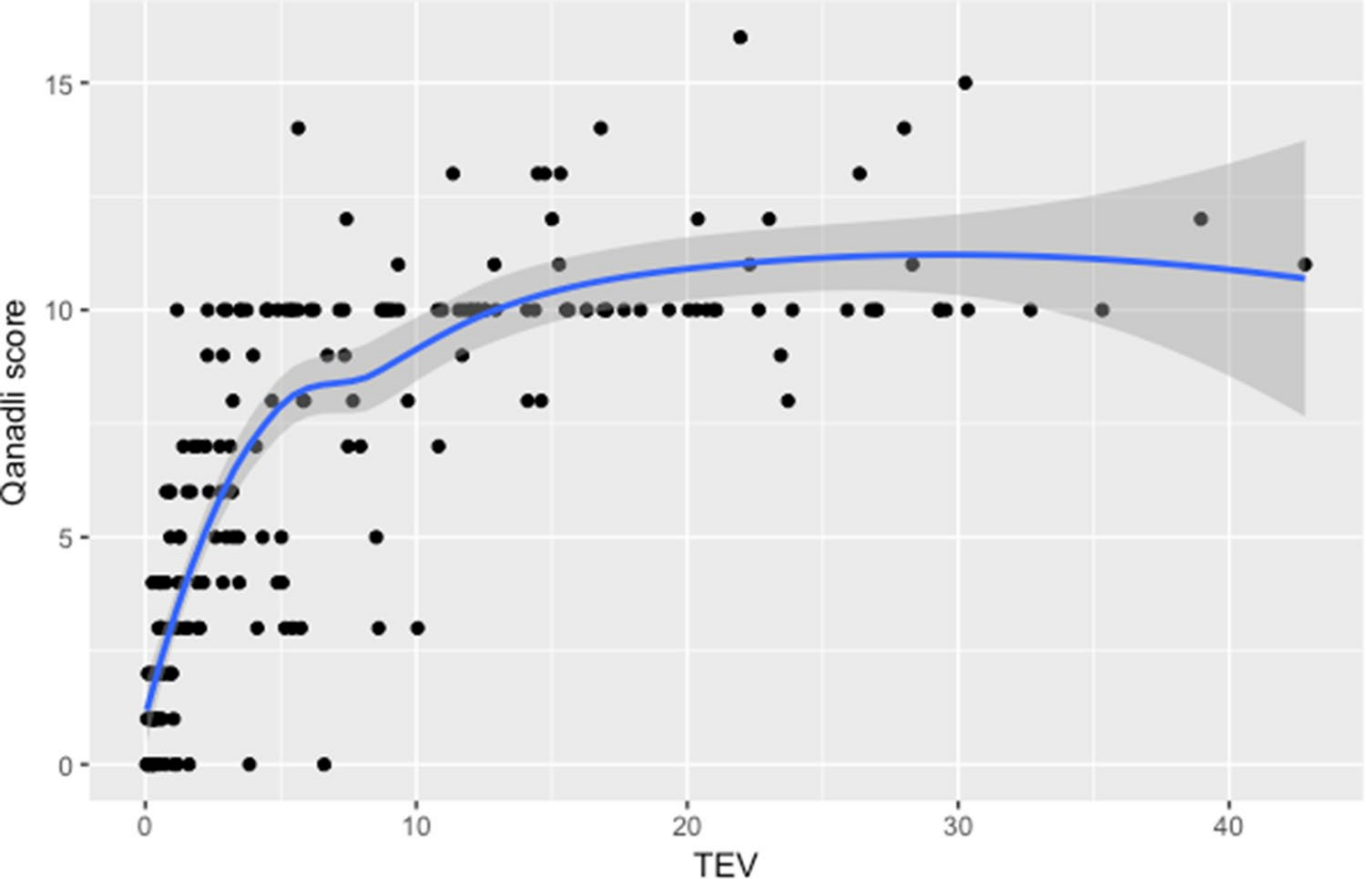
TEV V.S. Qanadli score. The correlation of the TEV and Qanadli score (r = 0.69, *p* < 0.001). However, the correlation did not seem to be linear. The regression line was drawn using the LOESS method (Local Polynomial Regression Fitting).

**Figure 2:**
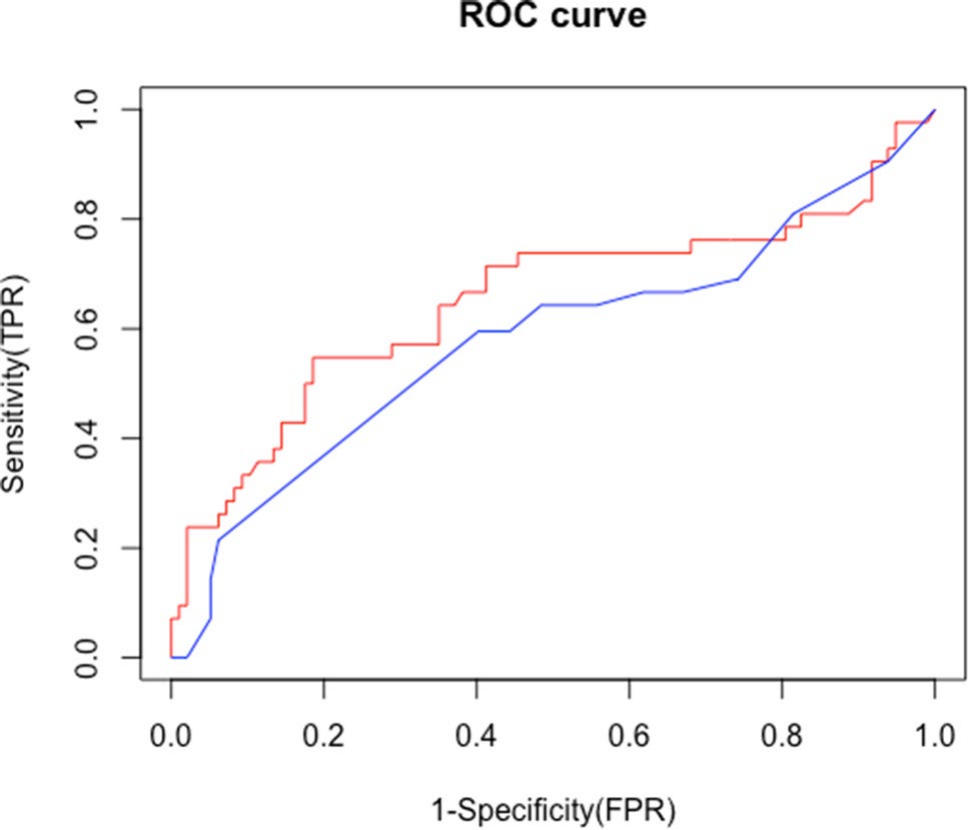
ROC curve to predict RV dysfunction. (Red: total emboli volume, blue: Qanadli score) (AUC: 0.65 vs. 0.58, *p* = 0.015).

TEV and the Qanadli score were strongly correlated (r = 0.69, *p* < 0.001; Fig. 1), although the correlation was stronger when TEV was < 10 cm^3^ and weaker when TEV was > 10 cm^3^ (Fig. 1). In addition, TEV was a better predictor of RV dysfunction than the Qanadli score, according to the ROC analysis (AUC: 0.65 vs. 0.58, *p* = 0.015; Fig. 2). Reproducibility, as evaluated using the intraclass correlation coefficient (ICC), was higher with TEV than with the Qanadli score. The ICCs of TEV and the Qanadli score were 0.99 (95% CI: 0.98–0.99) and 0.74 (95% CI: 0.58–0.85), respectively.

**Figure 3:**
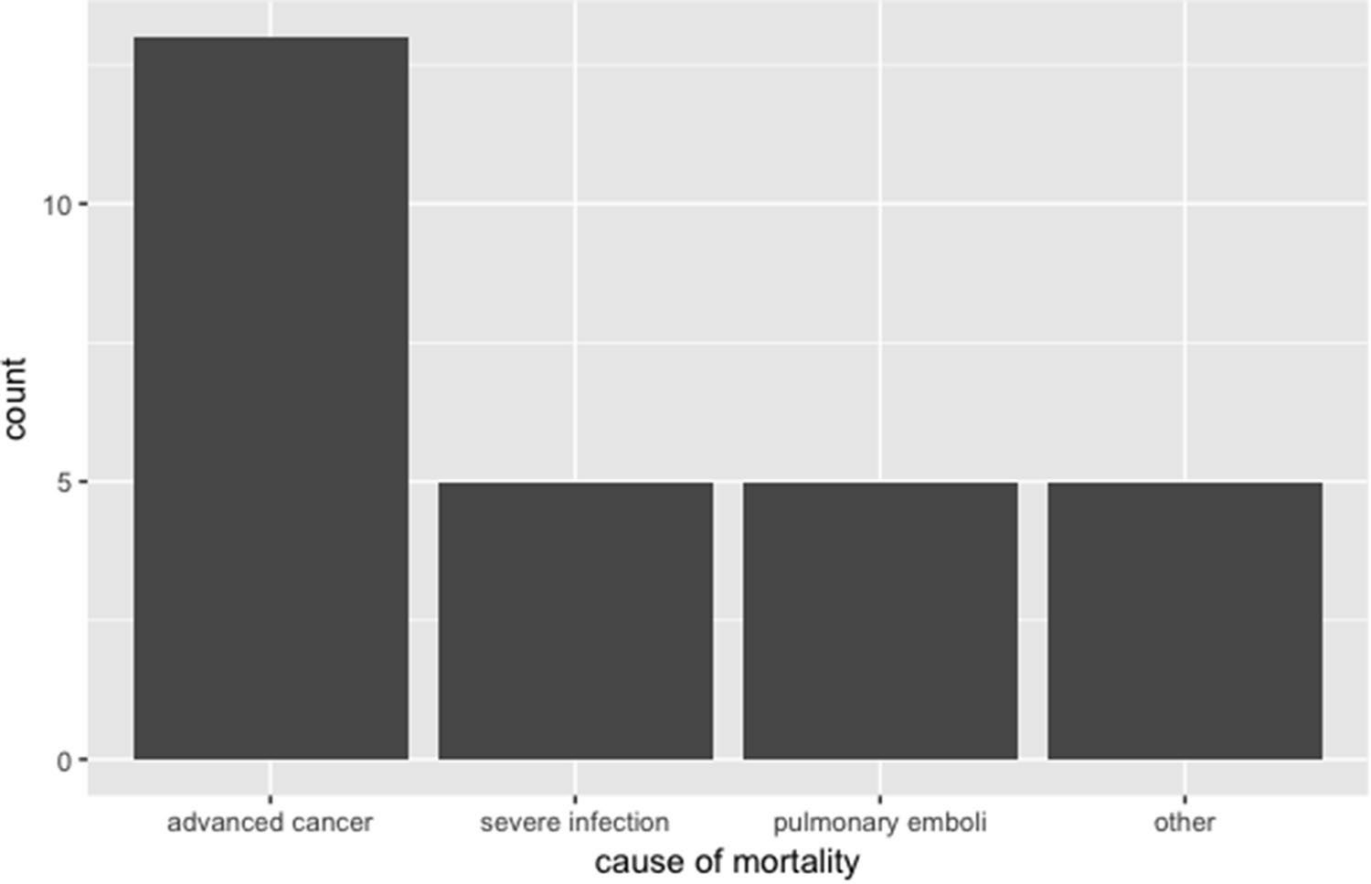
Cause of mortality. Other: stroke, acute myocardial infarction, COPD, ESRD with hyperkalemia.

The 30-day, 90-day, 1-year and 2-year mortality rates were 8.5% (17 cases), 13.9% (28 cases), 24.4% (49 cases) and 29.9% (60 cases), respectively, and these were not significantly correlated to TEV or the Qanadli score. The leading cause of death was cancer (Fig. 3). To evaluate the relationships between radiologic factors and impending shock, we assessed the correlation between impending shock and the echocardiogram findings (sPAP and RV dysfunction) and CT measurements (TEV, Qanadli score, RV diameter, and RV/LV ratio). TEV (OR = 1.86, 95% CI: 1.01–1.12 per 10 cm^3^ increment, *p* = 0.013) and the RV/LV ratio (OR = 2.51, 95% CI: 1.11–5.65, *p* = 0.02) were significantly correlated with impending shock (Table 6). The TEV and Qanadli score did not show significant difference in the prediction of impending shock according to the ROC analysis (AUC0.43 vs. 0.50, *p* = 0.58). Multivariate logistic regression analysis with backward selection was used to identify the factors related to impending shock. In addition, since the patients with cancer had poorer outcomes in our study (Fig. 3), they were excluded from further analysis of shock. The clinical (chronic heart lung disease, age, gender, BNP, Troponin I, systolic blood pressure and respiratory rate) and radiological (TEV, RV/LV ratio, MPA, MPA/AAO (ascending aorta)) factors were included as the factors in the model selection. The final model included TEV, age, respiratory rate and MPA, and showed that TEV (OR: 2.28 per 10 cm^3^ increment, *p* = 0.007), age (OR: 1.06, *p* = 0.012), and respiratory rate (OR = 1.23, *p* = 0.001) were significantly related to impending shock (Table 5).

**Table 5:** Backward stepwise logistic regression for impending shock.

	OR	95% CI	*p*-value
Total emboli volume (unit of 10 cm^3^)	2.28	1.27–4.29	0.007*
Age	1.06	1.02–1.12	0.012*
Respiratory rate	1.23	1.10–1.42	0.001*
MPA	0.76	0.16–3.37	0.72

The Full model includes total emboli volume, RV/LV ratio, MPA, MPA/AAO, chronic heart lung disease, age, gender, BNP, Troponin I, systolic blood pressure and respiratory rate as the factors. After backward stepwise selection, the remaining factors were total emboli volume, age, respiratory rate, and MPA.

*BNP* B-type natriuretic peptide, *RV* right ventricle, *LV* left ventricle, *MPA* diameter of main pulmonary artery, *AAO* ascending aorta. * The threshold for statistical significance was *p* < 0.05.

## Discussion

In this study, we described a CT volumetric quantification method for TEV in acute PE, and we found that it was independently associated with the Qanadli score, echocardiographic findings (sPAP and RV dysfunction), SpO_2_, and troponin I. Most of these measurements are commonly available and can be obtained from the emergency department. Furthermore, we noticed that RV dysfunction, as assessed using echocardiography, is strongly correlated with TEV but not the Qanadli score, and that CT 3D measurements of clot burden played an important role in predicting impending shock but not short-term mortality.

Historically, MDCT has been considered the gold standard for diagnosing patients with acute PE^16^. However, it has played a limited role in prognostication. In the current clinical guidelines issued by the AHA and ESC, a high RV/LV ratio is one of the CT factors associated with poor prognosis^2,3^. A meta-analysis showed that a RV/LV ratio > 1.0 on CT was associated with a 2.5-fold increased risk for all-cause mortality (OR: 2.5, 95% CI: 1.8–3.5)^17^. In our study, we also found that an increased RV/LV ratio was significantly associated with an increased risk for impending shock. Conversely, TEV may be an important predictor for stratifying acute PE patients who are referred to the emergency department. Increases in sPAP were correlated with increasing TEV quartiles (Table 3) and a similar pattern was seen with RV dysfunction and troponin I, while the reverse pattern was observed with SpO_2_. We also found that of the MDCT and echocardiogram findings in patients without cancer, TEV and RV/LV ratio were significantly correlated with impending shock (Table 6). Furthermore, in the backward stepwise logistic regression analysis, TEV, age, and respiratory rate were independently associated with impending shock (Table 5). The presence of shock is clinically relevant to the management and prognosis of acute PE. Approximately 5% of intermediate-risk PE patients who initially show hemodynamic stability will develop hemodynamic decompensation within the first 48 h and up to as late as five days later^18^. Traditionally, for acute PE patients, the presence of RV dysfunction on echocardiography and the RV/LV ratio measured via CT are key indicators for shock. Our study suggests that precisely measured 3D embolic volume in the pulmonary arteries may be a more accurate predictor of impending shock than these two indicators. To date, there are limited data on the clinical implications of embolic burden. Herein, we have introduced the clinical feasibility of this novel imaging modality in PE patients referred to the emergency department. We propose that its use for the timely diagnosis of PE and stratification of risk for impending shock in such patients may provide valuable information in acute settings by indicating when urgent thrombolysis or thrombectomy interventions are warranted.

**Table 6:** Different image modality for predicting impending shock.

Factors	OR	95% CI	*p*
Total emboli volume (unit of 10cm^3^)	1.86	1.13–3.05	0.013*
Qanadli score	1.09	0.95–1.26	0.22
RV dysfunction (Yes vs No)	1.25	0.30–4.70	0.74
sPAP	1.007	0.97–1.04	0.71
RV/LV ratio	2.51	1.11–5.65	0.02*
Diameter of RV	1.76	0.94–3.36	0.08
MPA/AAO	0.96	0.06–14.6	0.98

Shock is defined as hypotension requiring vasopressor (including dopamine, norepinephrine and vasopressin).

*BNP* B-type natriuretic peptide, *RV* right ventricle, *LV* left ventricle, *MPA* diameter of main pulmonary artery, *AAO* ascending aorta. * The threshold for statistical significance was *p *< 0.05.

For echo-based RV dysfunction (Table 3), Q2 (6.06%) is smaller than Q1 (27.8%). After excluding counts with only RV dilatation (diameter < 30 mm), which is weak evidence of RV dysfunction, we found that the percentages of Q1 and Q2 were equal (4%). This result supports other data (sPAP, RV diameter, RV/LV) that show that when the embolic burden is small (maximal TEV in Q2 is < 4 cm^3^), changes in cardiac morphology and function are minor. To our knowledge, no other studies prove the relationship between cardiac function and precise embolic burden, and we offer this perspective to clarify the relationship.

Compared to the Qanadli score, TEV not only more accurately assesses 3D emboli burden but is also better correlated with RV dilatation and echo-based RV dysfunction. Increasing TEV and Qanadli score can both indicate a larger clot burden in the pulmonary arterial circulation, which can lead to pressure overload and RV dilatation. Recently, a small study with fifty-eight patients with acute pulmonary embolism showed that quantitative volumetric measures of TEV was positive correlation with RV/LV ratio^19^. In our study, TEV and the Qanadli score were well correlated, especially in the case of main pulmonary arterial involvement. However, the Qanadli score only assigns one point to each subsegmental embolus, irrespective of its length and the number of related subsegmental arteries. Therefore, this score cannot reflect the actual volume of the clot burden. Indeed, we showed that the Qanadli score is only well correlated with a smaller TEV (Fig. 1), due to this underestimation. Furthermore, as a semiquantitative method, the Qanadli score is difficult to calculate and has low reproducibility and high interobserver variability^20^. Here, we have demonstrated that TEV measurement, which is both semiautomatic and fully quantitative, has better reproducibility than the Qanadli score (ICC: 0.99 vs. 0.74, respectively).

In this study, there was no significant association between TEV and 30- and 90-day mortality rates, but this was not unexpected. There are only two small studies published before 2010 that have shown a significant association between semiquantitative clot burden assessment and short-term survival^11,21^, whereas several more recent studies with a large number of subjects have failed to show a significant association between the Qanadli score and short-term survival^22,23^. This may be due to improvements in the early diagnosis and treatment of patients with shock and cardiac arrest from acute PE, which has increased their overall survival rate. Recently, Stein et al. investigated the mortality of PE patients in the United States and showed that, from 1999 to 2017, the mortality of all high-risk patients decreased from 72.7 to 49.8%^24^. A decreasing mortality rate for acute PE makes demonstrating a significant correlation between radiologic markers and mortality more difficult. This could explain why, despite 79.3% of our patients having a high-risk sPESI score, TEV was not significantly associated with short-term mortality. In contrast, cancer, which is a major risk factor for PE and is used in calculating the sPESI score, was the leading cause of death in our patients (Fig. 3). Similarly, other PE risk factors, such as advanced age and certain medical comorbidities^25,26^ can significantly affect morbidity and mortality rates, even in patients who are considered to have a low-risk PE because they are normotensive with normal biomarker levels and no RV dysfunction on imaging^20^. Therefore, to more comprehensively stratify the severity of acute PE and the risk of early (in-hospital or 30-day) death, additional factors such as hemodynamic status, clinical condition, RV dysfunction, and troponin I levels are important and need to be explored^24^.

Our study has some limitations. First, it is a retrospective study with an exclusively Asian population from a single medical center, which may represent a selection bias and limit generalizability. Second, our 3D CT volumetric method is semiautomatic, still requires manual adjustments and doesn’t compared to other vendor-independent softwares. Recently, a fewstudies used artificial intelligence (AI) for training the data augmentation and fully automatic quantification of embolic burden. Roman et al.^27^ using deep learning with realistic data augmentation showed good results in PA segmentation compared to semi-automatic quantification ground truths. Liu el al demonstrated the good correlation between deep leaning convolutional neural network full quantitative embolic burden and the Quanadli, Mastora scores in acute pulmonary emblism, but no further investigation into laboratory data and echocardiographic findings^28^. Future large cohort studies compares quantitative embolic burden among different semi-automatic and AI softwares may help to improve clinical efficiency by generating 3D volumetric results before radiologist interpretation and reducing possible manual operator errors.

In conclusion, our study demonstrated that a 3D CT method for quantifying acute pulmonary embolism provides results that are significantly linked to clinical condition, laboratory data, RV dysfunction, and impending shock. 3D CT measurements of clot burden may thus become a useful and feasible method for acute PE risk stratification and prognostication.

## Methods

**Figure 4:**
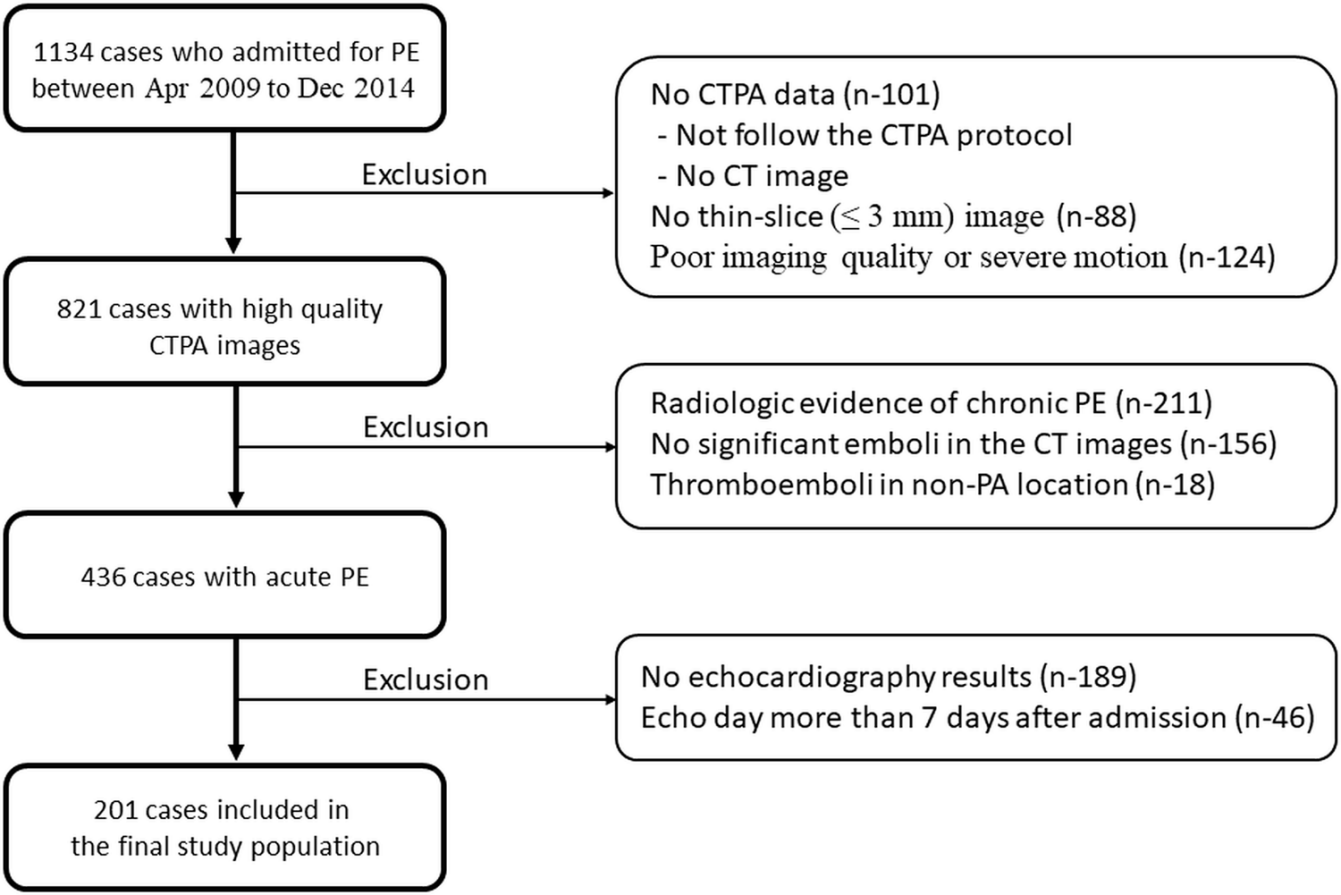
Flow chart of enrolled participants meet the requirements.

This retrospective study was approved, and all methods were performed in accordance with the relevant guidelines and regulations by the institutional review board of Mackay Memorial Hospital (no. 19MMHIS293e), Taipei, Taiwan. From April 2009 to December 2014, 1134 patients with either a clinical or a radiologic diagnosis of acute PE were evaluated. The medical records and images for all cases were reviewed by two radiologists (W.H. and C.Y.), and all images have been anonymous. A total of 201 patients were included in the final analysis after the following exclusion criteria had been applied: (1) no CT angiography for PE (i.e., they either had a CT scan that did not follow the pulmonary artery protocol or they did not have any CT imaging data); (2) no thin-slice (≤ 3 mm) image reconstruction; (3) poor imaging quality; (4) radiologic evidence of chronic PE; (5) no significant emboli detected in the CT images; (6) thromboemboli in non-pulmonary vascular locations (e.g., venous emboli or left atrial thrombus); or (7) no echocardiography results; (8) echocardiography performed more than 7 days after admission (Fig. 4)..

### Clinical information

Patient characteristics (age, gender, and history of cancer and chronic heart lung disease), their clinical presentation data (blood pressure, oxygen saturation [SpO_2_], and heart rate) and cardiac biomarkers (D-dimer, B-type natriuretic peptide [BNP], and troponin I) were recorded. The simplified Pulmonary Embolism Severity Index (sPESI)^29^ was calculated. To evaluate the effect of embolic volume on RV function, we collected the following echocardiographic data: estimated systolic pulmonary artery pressure (sPAP); RV and left ventricle (LV) diameter; and presence of RV dysfunction. RV dysfunction was diagnosed if the echocardiogram showed any of the following morphological remodeling features: (1) morphological segmental RV abnormality (e.g., presence of akinesia of the mid-free RV wall with preserved apical contractility, as indicated by McConnell’s sign) with RV diameter ≤ 30 mm; (2) RV/LV diameter ratio > 1; or (3) RV diameter > 30 mm^30–32^. To evaluate clinical prognosis, we recorded the presence of shock (defined as requiring vasopressors) at emergency-department presentation or during the course of hospitalization, 30- and 90-day mortality, and the leading cause of mortality.

### The CT pulmonary angiography (CTPA) protocol

All CTPA studies were performed with a 16-slice (Somatom Sensation 16, Siemens Healthcare, Forchheim, Germany) or a 64-slice (Aquilion-64, Toshiba Medical Systems, Otawara, Japan) MDCT scanner. The standard CTPA for PE was performed according to the following scan and reconstruction protocols: scans were acquired within a single breath-hold and obtained with a detector width of 16 × 0.672 mm or 64 × 0.5 mm, a tube voltage of 120 kVp, automatic exposure control (AEC) for the tube current, a 0.5 s gantry rotation time, and a 2 mm reconstructed slice thickness. The injection rate of the contrast medium, which was 80 mL of intravenous iohexol (Omnipaque-300) or iopromide (Ultravist-300), was 2.5–3 mL/s. Scans were performed using a bolus-tracking technique and initiated when the contrast medium was first seen in the pulmonary trunk after injection. The scan covered the region from the lung apex to the lowest hemi-diaphragm.

### Image analysis and 3D emboli segmentation

All CTPA images were reviewed by a radiologist (W.H.) with eight years of experience in chest CT. Any indeterminate image interpretation was resolved after discussion and review with a senior radiologist (C.Y.) with 18 years of experience in chest CT. For each case, the PE was identified and defined as an intraluminal central filling defect with partial or complete occlusion of the pulmonary artery. Cases with poor imaging quality (motion artifact, high noise, or poor pulmonary artery enhancement) were excluded.

The Qanadli score^10^ was used for semiquantitative calculation of clot burden. Using the Qanadli score, the arterial tree of each lung was divided into 10 segmental arteries (three to the upper lobes, two to the middle and lingular lobes, and five to the lower lobes). The presence of an embolus in a segmental artery was scored 1 point and the proximal arterial level was scored points equal to the sum of the distal segmental arteries. To indicate the severity of the embolus obstruction, the following weighting factor was used: 2 for total occlusion, 1 for partial occlusion, and 0 for no thrombus observed. The maximal Qanadli score for one patient was therefore 40.

**Figure 5:**
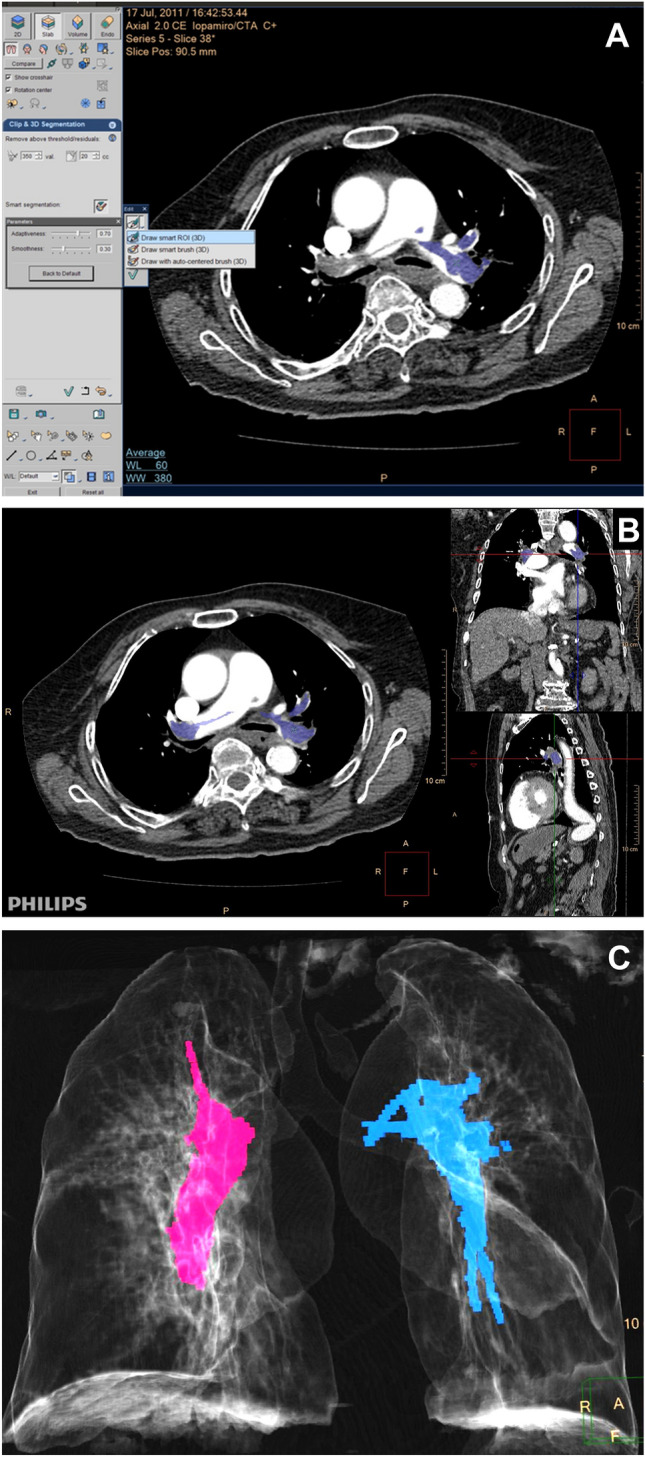
(**A**) 3D segmentation method (Draw smart ROI) was used to semi-automatically measure the volume of thrombus. (**B**) The boundaries of thrombus was checked visually on axial, coronal, and sagittal planes. (**C**) A 75-year-old female with high D-dimer (7898 mg/l) and mild elevated Troponin-I (0.64 ng/ml). 3D CT image quantification revealed large emboli burden in right pulmonary artery (14.3 cm^3^, pink color) and left pulmonary artery (15.03 cm^3^, blue color).

A 3D-based CT method was used for absolute quantitative evaluation of the total embolic volume (TEV). CTPA images were transferred to a dedicated workstation (IntelliSpace Portal [ISP] 9.0, Philips Medical Systems Nederland) and the embolic volume was measured semiautomatically. Filling defects were visually identified within the arterial tree (including pulmonary trunk, right and left pulmonary arteries, to the level of segmental arteries), and the boundaries of the filling defects were defined semi-automatically by the workstation using 3D segmentation method known as Draw smart ROI (Figure 5A) and visually check on axial, coronal, and sagittal planes (Figure 5B). The workstation also automatically interpolated the filling defect between the imaging slices and then calculated the TEV (Figure 5C) Other CT parameters, such as diameter of the main pulmonary artery (MPA), RV, and LV, were also measured, and the RV/LV ratio was calculated. RV and LV diameters were measured on the axial image between the inner surface of the free wall and the surface of the interventricular septum.

### Clinical endpoint

To evaluate clinical prognosis, we prespecified and recorded the clinical presentation of shock (defined as requiring vasopressors) at emergency-department presentation as the primary endpoint. We further assessed 30- day and 90-day, 1-year and 2-year all-cause mortality and rehospitalization during subsequent follow-up.

### Statistical analysis

Statistical analysis was performed using R (version 3.2.3; http://www.r-project.org/). Quartiles were used to display the pattern of increasing TEV and its effects on cardiac echo, CT, Qanadli score, and lab data findings. A multivariate logistic regression model with backward stepwise selection was used to evaluate the factors affecting RV dysfunction and shock. The correlations between the different image modalities—CT (TEV, Qanadli score, RV/LV ratio, RV diameter) and cardiac echography (sPAP, presence of RV dysfunction)—and impending shock were evaluated using odds ratios (OR). Receiver operating curve (ROC) analysis was used to compare the ability of TEV and Qanadli scores to predict RV dysfunction. The threshold for statistical significance was *p* < 0.05.

### Reproducibility

TEV and Qanadli scores were independently measured and calculated for a random sample of 50 cases generated from the final analysis set by two experienced observers (C.Y. and W.H.) blinded to the initial results. The intraclass correlation coefficient was used for analysis.

### Ethics approval and consent to participate

Study approval was obtained from the Mackay Memorial Hospital Institutional Review Board (IRB no. 19MMHIS293e). This 3D CT image was reconstructed from de-identified data and the informed consent was waived during Mackay Memorial Hospital Institutional Review Board review.
